# Use of Purastat, a novel haemostatic matrix based on self-assembling peptides in the prevention of nasopharyngeal adhesion formation

**DOI:** 10.1016/j.ijscr.2020.04.027

**Published:** 2020-05-08

**Authors:** Eugene Wong, Joyce Ho, Murray Smith, Niranjan Sritharan, Faruque Riffat, Mark C. Smith

**Affiliations:** a Department of Otolaryngology, Head and Neck Surgery, Westmead Hospital, Westmead, NSW, Australia; b Sydney Medical School, University of Sydney, Camperdown, NSW, Australia

**Keywords:** Nasopharyngeal stenosis, Purastat, Adhesion, Haemostasis

## Abstract

**Introduction:**

Purastat is a novel haemostatic agent that has recently been used effectively in nasal procedures, specifically in functional endoscopic endonasal surgery in the prevention of post-operative epistaxis while minimising adhesion formation. This study presents a case of successful application of Purastat following division of a nasopharyngeal/palatal stenosis following radiotherapy.

**Presentation of case:**

A 49-year-old male developed severe nasopharyngeal stenosis following concurrent chemoradiotherapy with curative intent a HPV positive base of tongue squamous cell carcinoma. Following treatment, while the patient experienced excellent metabolic response, he began to progressive nasal obstruction which he reported to be significantly affecting his quality of life. Following multidisciplinary team discussion, division of the stenosis was considered appropriate.

Under general anaesthesia, the patient was positioned lying supine with the neck extended. A Boyle-Davis gag with lip and teeth protection was placed and a Y-suction catheter inserted to lift the palate from the posterior pharyngeal wall. Coblation using a PDW wand was used to divide the area of fibrosis bilaterally toward the superior tonsillar pillar until palatopharyngeus musculature was visible. Injection of 4 mg of Dexamethasone with a hypodermic needle was then performed followed by application of 4 ml of topical Purastat onto the raw surfaces after haemostasis was confirmed.

The patient was discharged the same day on simple analgesia and instructions to avoid exertion for two weeks. He attended follow-up in the outpatient department two months later and reported persisting resolution of his nasal obstructive symptoms. No evidence of recurrence or residual adhesion tissue was noted.

## Highlights

• Purastat is a novel haemostatic agent used effectively in nasal procedures in the prevention of epistaxis and adhesions.
• This study describes the use of PuraStat following division of nasopharyngeal stenosis to reduce the risk of restenosis.
• On last follow-up the patient experienced excellent resolution of symptoms with no evidence of readhesion.
• The authors propose that PuraStat may be used in other surgical interventions to prevent mucosal adhesion formation.

## 1 Introduction

Nasopharyngeal stenosis (NPS) has been defined as “a subtotal obliteration of the normal space behind the posterior pillars and the soft palate caused by adhesive bands of scar tissue between these structures and the posterior pharyngeal wall”. Acquired NPS [1] is a rare complication of chemoradiation therapy for head and neck cancers. It is also a known complication of the commonly performed tonsillectomy and adenoidectomy. Other causes of NPS include maxillofacial trauma and infections such as diphtheria, syphilis, and tuberculosis [2]. NPS can result in nasal obstruction and compromise nasal breathing. Other sequelae of this complication include hyponasality, rhinorrhoea, dysphagia, anosmia, and obstructive sleep apnoea [3].

A variety of procedures exist within the literature to repair NPS. These include palatal transposition flaps [1,4,5], surgical release of adhesions using a CO2 laser or plasma radiofrequency-based coblation [6], the use of a customised nasopharyngeal obturator [2,[7], [8], [9], [10]], endoscopic-guided placement of a silastic stent [3], topical mitomycin-C application [11], balloon dilation [12], radial forearm free flap reconstruction [13], and even the use of transoral robotic surgery to create a modified pharyngeal flap [14].

Nevertheless the goal of all of these surgical procedures remains consistent - to open the stenotic segment and maintain its patency for as long as possible. A significant concern with regard to any surgical intervention for NPS, however, remains the possibility of restenosis and therefore future revision surgery. As a result, several techniques are often pursued to prevent restenosis, including steroid injections and the insertion of stents.

PuraStat® (3-D Matrix Ltd, Tokyo) is a synthetic haemostatic material which comes in a prefilled syringe [15]. It is composed of three types of amino acids that bond together to form a peptide. Upon a change in pH caused by exposure to an ionic solution such as blood, the peptides self-assemble to form a three-dimensional nanofiber scaffold which mimics human extra-cellular matrix. This matrix sticks to and seals the blood vessel, allowing for haemostatic control with a mechanical barrier [16]. PuraStat® is indicated for haemostasis during surgery, in the event of bleeding from small blood vessels or capillaries of the parenchyma of solid organs and the gastrointestinal tract and oozing from vascular anastomoses [15].

The use of Purastat has recently gained popularity in otolaryngology, particularly in endoscopic endonasal surgery (EES). In this setting, the agent has been used on raw mucosal surfaces, such as those produced following powered inferior turbinoplasty or sinus surgery primarily to achieve haemostasis, without causing the detrimental effects of other haemostatic techniques such as nasal packing tamponade or the tendency for synechiae formation following other haemostatic matrix agents in the literature.

Lee et al’s case series demonstrated that in a cohort of 60 consecutive patients who underwent powered turbinoplasty followed by topical application of Purastat, no patients experienced rebleeding or evidence of adhesion formation at 4 weeks of post-operative follow-up.

Therefore, Purastat was trialled in a patient aiming to reduce both rebleeding rates and the rate of adhesion formation and restenosis following surgery for NPS. This report details the author’s experience in the use of this agent for this indication and speculate on future potential applications where prevention of mucosal adhesions are desired. This work has been reported in line with the SCARE criteria [17].

## 2 Presentation of case

A 49-year-old male developed severe nasopharyngeal stenosis following concurrent chemoradiotherapy with curative intent for a HPV positive base of tongue squamous cell carcinoma. He had no other relevant past medical history prior to treatment. Following treatment, while the patient experienced excellent metabolic response, he began to progressive nasal obstruction which he reported to be significantly affecting his quality of life. Following multidisciplinary team discussion involving other otolaryngologists, general surgeons and allied health teams, division of the stenosis was considered appropriate.

Under general anaesthesia, the patient was positioned lying supine with the neck extended. A Boyle-Davis gag with lip and teeth protection was placed. A Y-suction catheter was inserted to lift the palate from the posterior pharyngeal wall under direct vision with 0 and 30° rigid nasendoscopes.

**Fig. 1:**
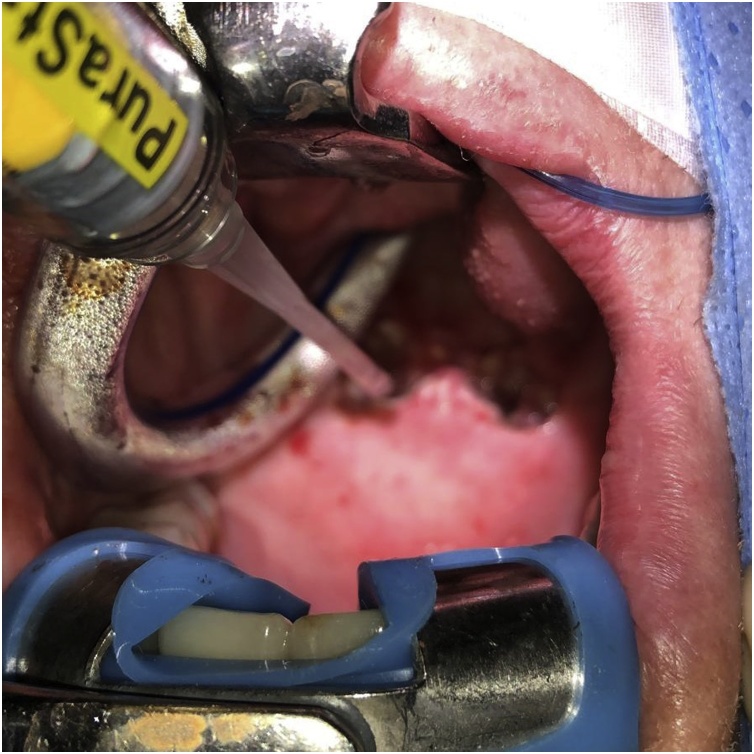
Intraoperative photograph during the application of PuraStat® to the raw mucosal surface. PuraStat® is applied using the provided prefilled syringe over the newly exposed raw mucosal surface following exposure of the pharynx using a Boyle-Davis mouth gag and coblation assisted division of the nasopharyngeal stenosis.

**Fig. 2:**
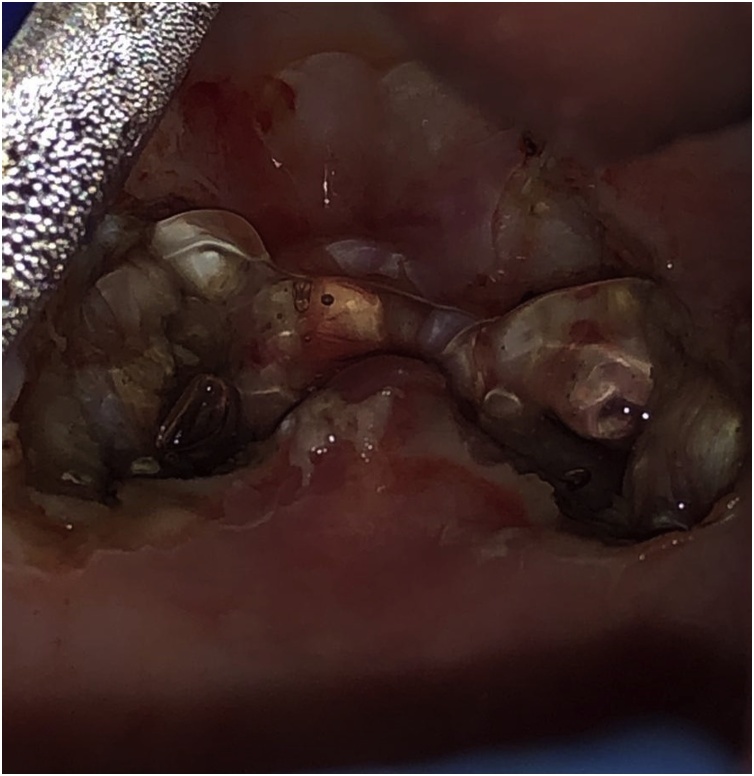
Intraoperative photograph following application of PuraStat®. PuraStat® has now been applied as a thin layer. The transparent property of the agent allows inspection for adequate haemostasis of the underlying tissues.

Coblation using a PDW wand was used to divide the area of fibrosis bilaterally. The wand was aimed laterally beginning from the midline toward the superior tonsillar pillar until the palatopharyngeus musculature was visible. Injection of 4 mg of Dexamethasone with a hypodermic needle was then performed bilaterally to the raw mucosal surface. Application of 4 ml of topical Purastat in a thin layer was subsequently performed onto the newly exposed surfaces (Fig. 1, Fig. 2). Haemostasis was confirmed prior to removal of the gag from the patient.

The patient was discharged the same day on simple analgesia, regular saline nasal rinsing four times per day and instructions to avoid exertion for two weeks. His diet was unrestricted. The patient experienced immediate improvement in subjective nasal patency post-operatively.

At two months follow-up in the outpatient department he reported complete resolution of his nasal obstructive symptoms. Oral examination using a headlight and flexible nasendoscopy at this stage demonstrated no evidence of recurrence or residual adhesion tissue was noted. The patient did not report any bleeding at last follow-up.

## 3 Discussion

The raw, injured mucosal surfaces that are exposed after surgical excision of scar bands in NPS can lead to further scarring and subsequent re-stenosis. As such, many techniques described in the literature are used to resurface the open areas and thereby prevent healing of the wound by secondary intention [13]. In addition to the aforementioned study in endoscopic endonasal surgery, a small volume of evidence exists in the literature demonstrating PuraStat’s effectiveness in reducing scar tissue formation.

In 2019, Oumrani et al examined the applicaton of PuraStat® in preventing post-operative oesophageal strictures in an animal model [18]. They performed circumferential endoscopic submucosal dissection on eleven pigs, and applied PuraStat® onto the oesophageal wounds of six models. At two weeks post-operatively, they found that two pigs in the treated group had asymptomatic oesophageal stricture on endoscopy, while all five pigs in the control group had oesophageal stricture and regurgitation seen on a barium oesophagram. They hypothesised that the use of the self-assembling peptide PuraStat® limited the scar tissue formation by promoting re-epithelialisation.

In this case report patient’s case, a combination of plasma radiofrequency coblation and PuraStat® haemostastic agent was used. Coblation technique meant that the amount of thermal damage to the surrounding tissues was limited, while the use of PuraStat® on the palate provided a biological wound dressing on the exposed submucosa, preventing excessive scar tissue formation. To the author’s knowledge, this is the first reported case of the use of an extracellular scaffold matrix for nasopharyngeal stenosis in humans.

Several limitations exist with this study. Primarily, it is limited by the short duration of follow-up. Ongoing, regular review will be required to ensure that the excellent symptomatic and objective result is maintained over time. Secondly, this patient developed NPS secondary to chemoradiation therapy, and therefore the technique may not necessarily be applicable to all aetiologies of NPS, such as iatrogenic causes from surgery such as adenotonsillectomy.

Nevertheless, several future directions can be considered following this study. This case report represents a successful proof of concept for the use of PuraStat in oral, nasopharyngeal and oropharyngeal mucosa, suggesting that further, higher powered observational and comparative studies are warranted to evaluate its effectiveness. Furthermore, adhesion formation (and bleeding) is a significant issue in several other anatomical regions of the upper aerodigestive tract, including laryngeal surgery, particularly at the anterior commissure, and the oropharynx –potential additional sites that may benefit from PuraStat application.

## 4 Conclusion

Nasopharyngeal stenosis is a rare complication of chemoradiation therapy in patients with head and neck malignancies. The scarring of NPS can also develop as a result of otolaryngological operations. This case report demonstrates effective use of a combination of coblation technology and PuraStat, a novel use synthetic self-assembling peptide, in minimising post-operative adhesion formation. Nevertheless, further studies need to be performed to assess the long-term outcomes of this technique.

## Declaration of Competing Interest

No conflicts of interest to declare.

## Funding

No sources of funding to declare.

## Ethical approval

This study is exempt from ethical approval as per the Western Sydney Local Health District Human Research Ethics Committee.

## Consent

Consent has been obtained from the patient.

## Author contribution

All authors EW, JH, MS, NS, FR, MCS contributed to study design, data collection, analysis and interpretation, manuscript preparation and approval of the final manuscript for submission.

## Registration of research studies

NA.

## Guarantor

Eugene Wong.

## Provenance and peer review

Not commissioned, externally peer-reviewed.
